# Microbiota-mediated effects of Parkinson’s disease medications on Parkinsonian non-motor symptoms in male transgenic mice

**DOI:** 10.1128/msphere.00379-23

**Published:** 2023-12-11

**Authors:** Nina Radisavljevic, Avril Metcalfe-Roach, Mihai Cirstea, M. Mahebali Tabusi, Tahereh Bozorgmehr, Haggai Bar-Yoseph, B. Brett Finlay

**Affiliations:** 1 Michael Smith Laboratories, University of British Columbia, Vancouver, British Columbia, Canada; 2 Department of Biochemistry and Molecular Biology, University of British Columbia, Vancouver, British Columbia, Canada; 3 Department of Microbiology and Immunology, University of British Columbia, Vancouver, British Columbia, Canada; University of Michigan-Ann Arbor, Ann Arbor, Michigan, USA

**Keywords:** gut microbiota, gut microbiome, neurodegenerative disease, Parkinson's disease, gut-brain axis, gut-microbiota-brain axis, animal models, mouse model

## Abstract

Parkinson’s disease (PD) is characterized by motor symptoms and a loss of dopaminergic neurons, as well as a variety of non-motor symptoms, including constipation, depression, and anxiety. Recently, evidence has also accumulated for a link between gut microbiota and PD. Most PD patients are on dopamine replacement therapy, primarily a combination of L-DOPA and carbidopa; however, the effect of these medications on the microbiota and non-motor symptoms in PD is still unclear. In this study, we explored the effects of chronic oral treatment with L-DOPA plus carbidopa (LDCD) on the gut microbiota and non-motor symptoms in males of a transgenic mouse model of PD (dbl-PAC-Tg(*SNCA*^A53T^);*Snca*-/-). To further test whether the effects of these PD medications were mediated by the gut microbiota, oral antibiotic treatment (Abx; vancomycin and neomycin) was included both with and without concurrent LDCD treatment. Post-treatment, the gastrointestinal, motor, and behavioral phenotypes were profiled, and fecal, ileal, and jejunal samples were analyzed for gut microbiota composition by 16S sequencing. LDCD treatment was found to improve symptoms of constipation and depression in this model, concurrent with increases in *Turicibacter* abundance in the ileum. Abx treatment worsened the symptoms of constipation, possibly through decreased levels of short-chain fatty acids and disrupted gut barrier function. LDCD + Abx treatment showed an interaction effect on behavioral symptoms that was also associated with ileal *Turicibacter* levels. This study demonstrates that, in a mouse model, PD medications and antibiotics affect PD-related non-motor symptoms potentially *via* the gut microbiota.

**IMPORTANCE:**

The motor symptoms of Parkinson’s disease (PD) are caused by a loss of dopamine-producing neurons and are commonly treated with dopamine replacement therapy (L-DOPA plus carbidopa). PD has also been associated with altered gut microbiota composition. However, the effects of these PD medications on PD-related non-motor symptoms and the gut microbiota have not been well characterized. This study uses a transgenic mouse model of PD to help resolve medication-induced microbiota alterations from those that are potentially disease relevant within a PD context, and explores how long-term treatment may interact with the gut microbiota to impact non-motor symptoms.

## INTRODUCTION

Parkinson’s disease (PD) is a neurodegenerative disorder that affects 1% of the population over 60 years of age (1). It is characterized by motor symptoms (tremor, rigidity, and slowed movement) caused by the loss of dopaminergic neurons within the substantia nigra and is associated with alpha-synuclein-containing Lewy body inclusions within remaining neurons. PD is also associated with multiple non-motor symptoms including constipation and mood disorders (2) which can have a significant impact on patients’ quality of life.

The neurodegeneration in PD is progressive and incurable with currently available therapeutics (2). PD treatments focus on symptom management with the “gold standard” treatment consisting of oral supplementation of L-DOPA (LD), a dopamine precursor able to cross the blood-brain barrier and replenish dopamine in the central nervous system (3, 4). In fact, LD responsiveness is often part of the criteria used for initial PD diagnosis (5, 6). LD is often co-administered with an aromatic L-amino acid decarboxylase (AADC) inhibitor, such as benserazide or carbidopa (CD), to prevent the premature conversion of LD to dopamine in the periphery (3, 4). Oral LD is absorbed primarily within the proximal small intestine (7), and PD-related gastrointestinal (GI) disturbances, particularly delayed gastric emptying, can have a profound impact on LD absorption and bioavailability (4, 7). Furthermore, LD has a short half-life in circulation and long-term use may lead to LD-induced dyskinesia (7).

LD is well known to effectively treat the motor symptoms of PD; however, its effect on non-motor symptoms—particularly gut motility and mood disorders—has not been as well characterized. Data on the effect of LD or LDCD treatment on cognition or mood in PD patients are limited, although side effects including worsened depression/anxiety, enhanced interest/reduced apathy, and improved memory have been reported (8, 9). LD treatment has been shown to delay gastric emptying time in healthy individuals (10–12) as well as in PD patients (13), perhaps through a direct effect on dopaminergic receptors within the stomach (14). LD treatment of mice during perinatal development led to increased exploratory behavior but no change to anxiety- or depression-like behavior (15). Since PD patients have been on medications for many years, the effect of these medications on non-motor aspects of the disease requires further investigation.

The GI comorbidities and findings of altered gut microbiota composition in PD patients have suggested that the gut-microbiota-brain axis may play a role in this disease (16). In support of this hypothesis, animal studies have indicated a causal role for the microbiota in PD pathophysiology (17) and alpha-synuclein deposits have been found within the enteric nervous system (ENS) of PD patients (18). Proposed mechanisms by which the gut environment may impact brain pathophysiology in PD include the following: the spread of misfolded alpha-synuclein up the vagus nerve, GI inflammation and a leaky gut barrier leading to systemic inflammation, and the production of neuroactive bacterial metabolites (16). Recently, the gut microbiota has come under investigation in terms of its impact on LD bioavailability (19, 20). Studies have shown that certain microbiota members can metabolize LD in a manner that is not inhibited by the AADC inhibitor, CD. Furthermore, the microbial LD metabolic capacity correlates with LD dose (20). Multiple studies have been performed that compare gut microbiota composition between PD patients and non-PD controls [reviewed in reference (21)]; only a few of these studies include PD patients who are treatment naïve, as LD treatment typically begins soon after diagnosis. Therefore, it may be important to identify the effect of PD medications on microbiota composition, to differentiate potentially disease-relevant microbiota alterations from those simply induced by disease treatment.

In this study, we investigated the effect of PD medications on non-motor PD-like symptoms and the gut microbiota within a transgenic mouse model of early PD (22). These mice display mild motor deficits, GI dysfunction, and behavioral alterations as early as 6 weeks of age (23). The study focused on chronic oral treatment with both LD and CD (LDCD) to best mimic the treatment regime of PD patients. Moreover, to test whether any impact of LDCD treatment is mediated by the gut microbiota, co-treatment with the antibiotics vancomycin and neomycin was included. Interestingly, we found that both LDCD treatment and antibiotic treatment—individually and in combination—affect PD-like non-motor symptoms and alter gut microbiota composition. In addition, the treatment effects observed on the PD-like phenotype of this mouse model appear to be associated with specific bacterial taxa.

## RESULTS

### PD medications and antibiotics, individually and in combination, affect the PD-like phenotype

**Fig 1:**
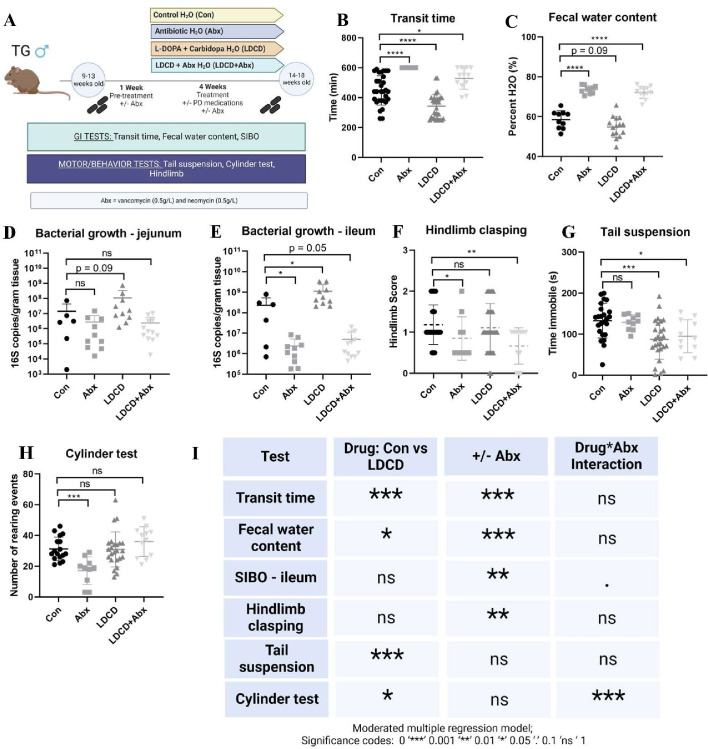
PD medications and antibiotics affect the PD phenotype in a transgenic mouse model. (**A**) Experimental design and timeline. (**B**) Whole-gut transit time. (**C**) Fecal water content. Bacterial levels in (**D**) jejunum and (**E**) ileum normalized to tissue weight (SIBO). (**F**) Hindlimb clasping reflex score. (**G**) Time immobile in tail suspension test. (**H**) Number of rearing events in cylinder test. (**I**) Moderated multiple regression analysis of effects of PD medications (Drug: Con vs LDCD) and antibiotics (+/− Abx), alone and in combination (Drug*Abx interaction) on phenotypic tests. All data are from post-treatment timepoint and obtained from 2 to 5 experimental replicates. Data points indicate individual mice; error bars indicate the standard deviation of the mean. *P < 0.05; **P < 0.01; ***P < 0.001; ****P < 0.0001 (by Mann-Whitney U test unless otherwise indicated). TG, transgenic; PD, Parkinson’s disease; Con, control; LDCD, L-DOPA plus carbidopa; Abx, antibiotics; SIBO, small intestinal bacterial overgrowth.

We assessed the GI, motor, and behavioral performance of PD mice treated with PD medications, antibiotics, or both using a variety of tests (Fig. 1A) and compared each of these treatment groups with control mice.

Mice that received chronic oral treatment with LDCD displayed decreased whole-gut transit time (*P* < 0.0001; Fig. 1B), indicating relief of the constipation-like phenotype exhibited by this mouse model. Although this group showed a trend for decreased fecal water content (*P* = 0.09; Fig. 1C), and slightly increased total bacterial load in the jejunum (*P* = 0.09; Fig. 1D) and ileum (*P* < 0.05; Fig. 1E), overall GI function seemed to be improved due to the strength of the effect on GI transit. LDCD-treated mice showed no improvements in striatal function compared to controls as measured by the hindlimb clasping test (Fig. 1F). However, LDCD treatment significantly decreased the time spent immobile during the tail suspension test (*P* < 0.001; Fig. 1G), representative of an improvement to the depression-like phenotype displayed by this model. The number of rearing events in the cylinder test—a measure of apathy-like symptoms or disinterest in exploring the environment—was unchanged in LDCD-treated mice when compared to control mice (Fig. 1H). A multiple regression analysis of the effect of LDCD treatment (independent of antibiotic treatment) revealed a significant effect of these PD medications on transit time, fecal water content, time immobile in the tail suspension test, and number of rearing events in the cylinder test (Fig. 1I). The cylinder test results were significant by the multiple regression model, but not in direct comparison to control mice, due to the slight trend toward an increased number of rearing events for both the LDCD and LDCD + Abx groups. In summary, these results indicate that treatment with LDCD improves PD-associated non-motor symptoms in this transgenic PD mouse model.

Chronic oral treatment with antibiotics (Abx) alone resulted in a significantly increased transit time (*P* < 0.0001; Fig. 1B) indicative of decreased gut motility; moreover, the concurrent increase in fecal water content (*P* < 0.0001; Fig. 1C) suggests a potential disruption in luminal solute transport, ultimately indicating that Abx treatment is linked to increased GI dysfunction. Total bacterial levels in the ileum (but not the jejunum) were also decreased due to antibiotic treatment (*P* < 0.05; Fig. 1D and E). Overall, Abx treatment appears to worsen the GI dysfunction present in this transgenic PD mouse model, due to the substantial increase in transit time. However, this treatment seemed to improve striatal function in this model, as shown by a lower hindlimb clasping score (*P* < 0.05; Fig. 1F). While these mice did not show any difference in time spent immobile in the tail suspension test (Fig. 1G), they did display a decreased number of rearing events in the cylinder test (*P* < 0.001; Fig. 1H) indicative of decreased interest in the environment and perhaps a worsened apathy-like phenotype. Multiple regression analysis of the effect of antibiotics (independent of PD medications) revealed a significant effect on transit time, fecal water content, small intestinal bacterial overgrowth (SIBO), and hindlimb clasping (Fig. 1I). Overall, these data indicate a potential beneficial effect of antibiotics on PD-associated striatal dysfunction, but a detrimental effect on PD-associated GI dysfunction and behavior in this transgenic mouse model.

To investigate the effect of combinatorial treatment with LDCD and Abx on phenotypic tests, a multiple regression analysis was performed to assess an interaction effect of both treatments, independent of each treatment alone (Fig. 1I). This revealed a significant treatment interaction toward improved performance in the cylinder test (*P* < 0.001). Trends toward an interaction effect were also observed in bacterial levels within the ileum. This suggests that modifying the gut microbiota (through Abx treatment) alters the behavioral effect of LDCD treatment.

### PD medications and antibiotics, individually and in combination, affect microbiota composition

**Fig 2:**
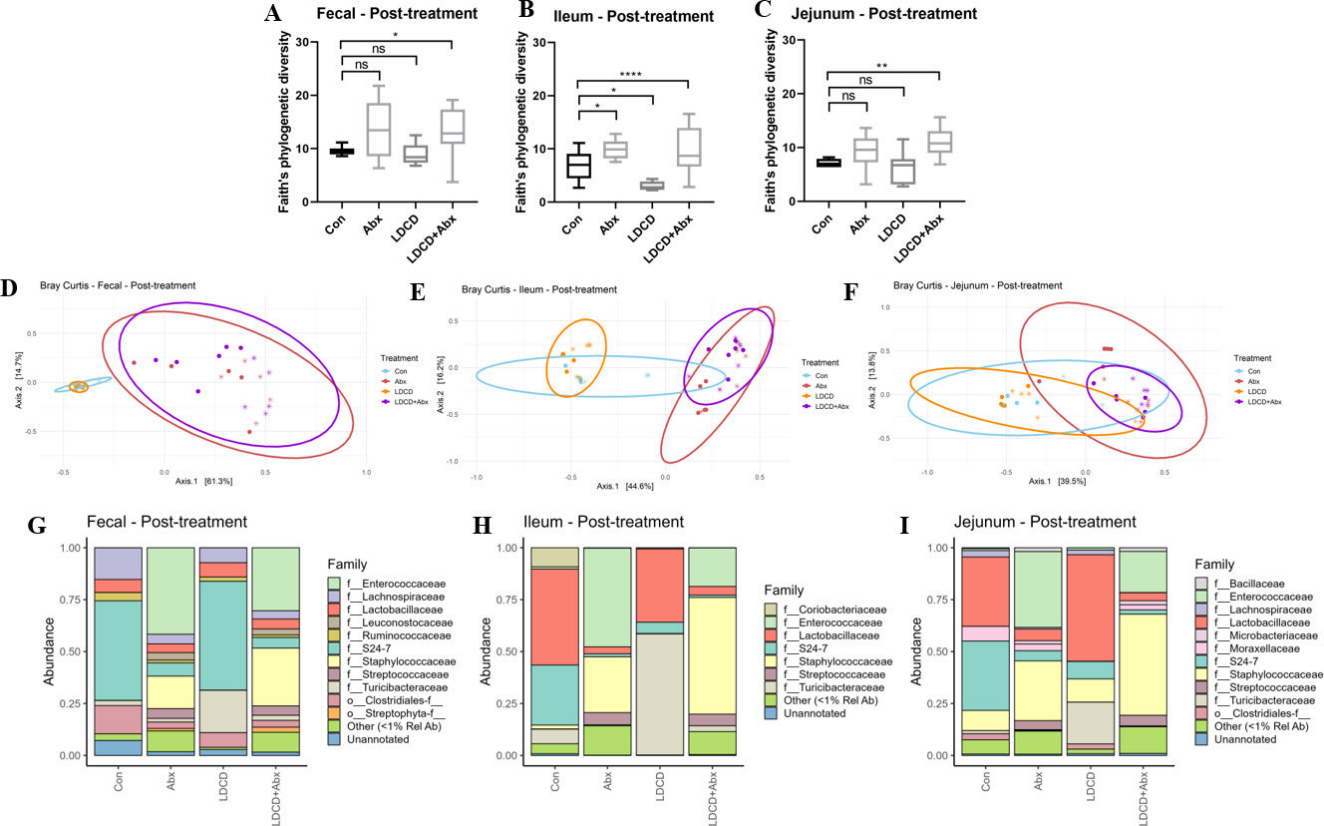
PD medications and antibiotics affect the microbiota in a transgenic mouse model. Alpha diversity—as measured by Faith’s phylogenetic diversity—of (**A**) fecal, (**B**) ileal, and (**C**) jejunal samples. Beta diversity—as measured by Bray-Curtis dissimilarity—of (**D**) fecal, (**E**) ileal, and (**F**) jejunal samples. Colors indicate different treatments, and shapes indicate different experimental replicates. Family-level average taxonomic abundance by treatment group in (**G**) fecal, (**H**) ileal, and (**I**) jejunal samples. All data are from post-treatment timepoint and obtained from 2 to 5 experimental replicates. Data points indicate individual mice; error bars indicate the standard deviation of the mean. *P < 0.05; **P < 0.01; ***P < 0.001; ****P < 0.0001 (by Mann-Whitney U test unless otherwise indicated). TG, transgenic; PD, Parkinson’s disease; Con, control; LDCD, L-DOPA plus carbidopa; Abx, antibiotics; SIBO, small intestinal bacterial overgrowth.

LDCD treatment altered the gut microbiota composition in multiple areas of the GI tract. Alpha diversity, as measured by Faith’s phylogenetic diversity, of the ileal microbiota community was significantly lower in LDCD-treated mice compared to controls (*P* < 0.05) but was not different in the jejunum or feces (Fig. 2A through C). This suggests that the most substantial LDCD-induced microbiota changes were occurring in the ileum, where LD is primarily absorbed. Between-group beta diversity (Bray-Curtis) analysis showed separation between control and LDCD-treated samples (Fig. 2D through F); permutational multivariate analysis of variance (PERMANOVA) testing revealed significant differences in fecal (*P*-adj <0.01) and ileal (*P*-adj <0.05) samples. PERMDISP analysis revealed no differences in dispersion between the groups.

The broad microbiota alterations induced by LDCD treatment are apparent in taxonomic differential abundance analysis (Fig. 2G through I). In fecal, ileal, and jejunal samples, the relative abundance of the bacterial family Turicibacteraceae appears to increase due to LDCD treatment. Furthermore, it is interesting to note that the control ileal and jejunal microbiota seems to be dominated by the families S24-7 and Lactobacillaceae; while Lactobacillaceae levels do not seem to change drastically upon LDCD treatment, the relative abundance of S24-7 seems to decrease in both GI sites. Overall, LDCD treatment induced microbiota alterations which will be discussed further below.

Chronic oral Abx treatment alone drastically shifted the gut microbiota composition in this transgenic PD mouse model (Fig. 2). Interestingly, Abx-treated mice showed increased alpha diversity (as measured by Faith’s phylogenetic diversity) in the ileum (*P* < 0.05; Fig. 2B) with this trend replicating in both fecal and jejunal samples (Fig. 2A and C). Bray-Curtis beta diversity PCoA plots show distinct clustering of Abx-treated mice, both with and without LDCD co-treatment, when compared to controls (Fig. 2D through F). PERMANOVA testing showed that Abx-treated mice had a distinct microbiota composition when compared to Con mice (*P*-adj <0.01 for fecal, ileal, and jejunal samples); PERMDISP analysis revealed no differences in dispersion between the groups except in fecal samples (Con vs Abx *P*-adj <0.05). Comparing family-level average taxonomic abundance between control and Abx-treated mice shows blooms of Enterococcaceae and Staphylococcaceae in all three gut regions sampled (Fig. 2G through I). Overall, these results show that antibiotic treatment resulted in broad shifts to the gut microbiota composition (rather than depletion), perhaps allowing the overgrowth of bacterial families with antibiotic resistance capabilities. We hypothesized that Abx treatment resulted in the depletion of dominant microbiota taxa, to allow for the growth of taxa that were below sequencing detection in control mice, leading to the increased alpha diversity observed.

Mice treated with both LDCD and antibiotics showed similar alpha diversity, beta diversity, and taxonomic abundances to mice treated with antibiotics alone (Fig. 2A through I) indicating that the effect of co-treatment on the microbiota is largely driven by antibiotics. However, beta diversity analysis showed that for ileal samples in particular, mice treated with both LDCD and Abx clustered separately from Abx-treated mice (PERMANOVA *P*-adj <0.05), with no differences in dispersion detected by PERMDISP analysis, demonstrating that this region may be a site of interaction between the two treatments.

### LD or CD individually have a minimal effect on the PD-like phenotype and gut microbiota

To investigate whether the beneficial effects of LDCD treatment on the PD-like phenotype were driven by either LD or CD treatment alone, a similar chronic oral treatment experiment was performed with each medication administered individually (Fig. S1A). While LD treatment slightly decreased transit time (*P* < 0.05; Fig. S1B), overall the effect of the medications in combination was not replicated by either medication alone. Similarly, the gut microbiota composition did not appear to be drastically altered, although some differences in alpha diversity were observed (Fig. S1G through I). Compared to controls, CD-treated mice (but not LD-treated mice) displayed significantly different clustering in fecal beta diversity analysis (PERMANOVA *P*-adj <0.05; Fig. S1J), although not to the same extent as with LDCD cotreatment; furthermore, this difference in beta diversity is potentially driven by differences in the dispersion of the groups (PERMDISP *P*-adj <0.05). These results imply that the beneficial effects of the medications on the GI and behavioral PD-like phenotype, as well as the distinct microbiota alterations, are dependent on the presence of both LD and CD together.

### LDCD-induced improvements to GI and behavioral PD-like phenotype may be linked to the bacterial genus *Turicibacter*

**Fig 3:**
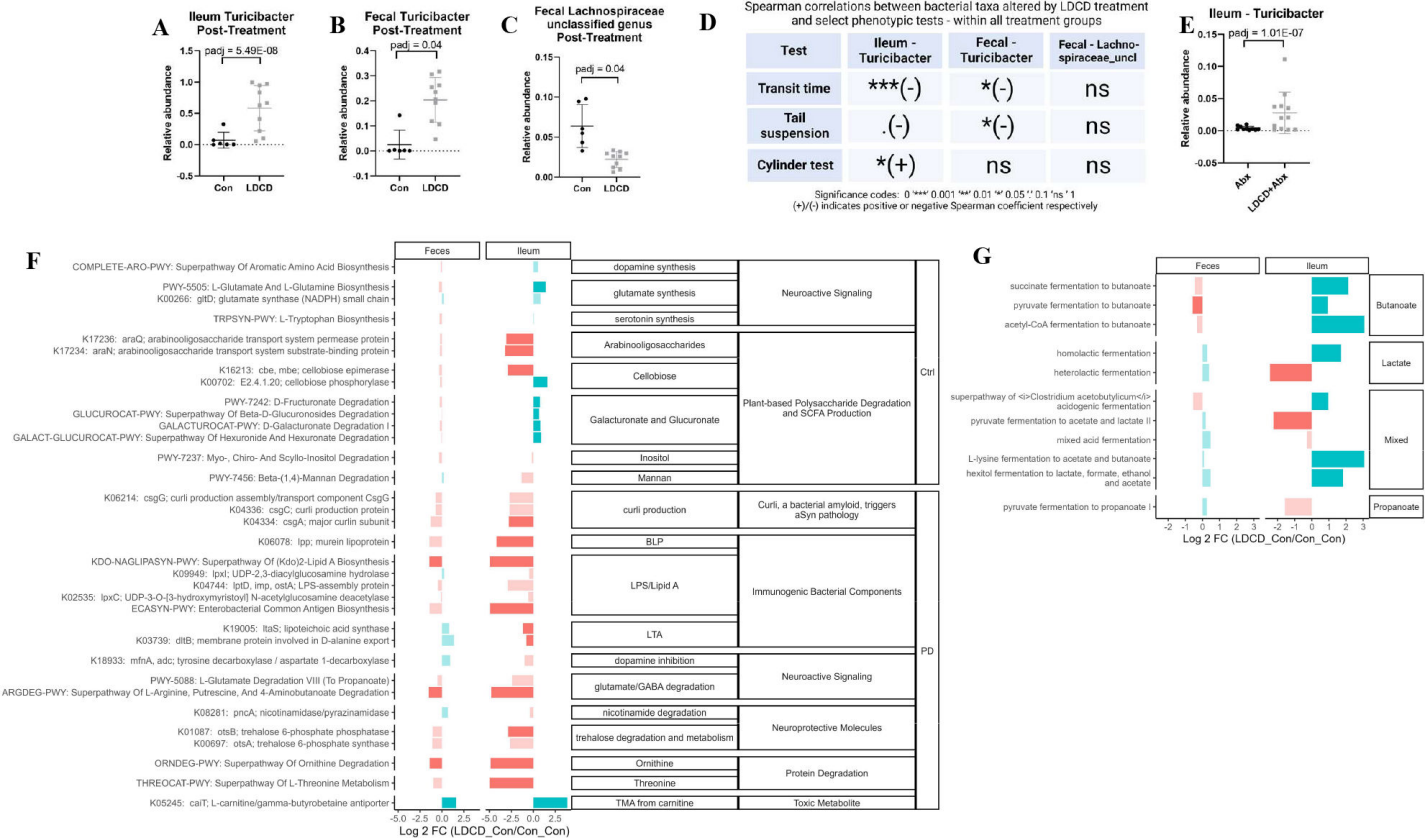
Turicibacter is linked to LDCD-induced improvements in gastrointestinal and behavioral phenotype in a PD mouse model. Relative abundance of bacterial genus Turicibacter in (**A**) ileal and (**B**) fecal samples post-treatment with LDCD. (**C**) Relative abundance of an unclassified genus in bacterial family Lachnospiraceae in fecal samples post-treatment. (**D**) Correlations between differentially abundant taxa and phenotypic tests (by Spearman correlation test, ± symbols indicate a positive or negative correlation, respectively). (**E**) Relative abundance of Turicibacter in the ileum of Abx and LDCD + Abx-treated mice. (**F**) PICRUSt2-predicted functional pathways in the feces and ileum of LDCD mice compared to controls; solid colored bars indicate significant hits (*P*-adj <0.01 and log 2 fold change > 2); blue bars indicate pathways increased in LDCD mice, red bars indicated pathways increased in Con mice. Pathways are grouped by their overarching function; the rightmost column denotes which pathways are associated with PD versus controls (Ctrl) by Wallen et al. (24). (**G**) SCFA-related PICRUst2-predicted functional pathways in the feces and ileum of LDCD mice compared to controls. All data are from post-treatment timepoint and two experimental replicates. Data points indicate individual mice; error bars indicate the standard deviation of the mean. *P < 0.05; **P < 0.01; ***P < 0.001; ****P < 0.0001 (by Mann-Whitney U test unless otherwise indicated). TG, transgenic; PD, Parkinson’s disease; Con, control; LDCD, L-DOPA plus carbidopa; Abx, antibiotics; SIBO, small intestinal bacterial overgrowth, SCFA, short-chain fatty acid; aSyn, alpha-synuclein; BLP, bacterial lipoprotein; LPS, lipopolysaccharide; LTA, lipoteichoic acid; GABA, γ-aminobutyric acid; TMA, trimethylamine.

We next investigated specific microbiota differences between control and LDCD-treated mice that could be linked to the disease-associated phenotypic improvements that we observed. On the genus level, only *Turicibacter* and an unclassified genus in the family Lachnospiraceae were significantly differentially abundant between these treatment groups. Ileal and fecal *Turicibacter* relative abundance was significantly increased in LDCD-treated mice (FDR *P* < 0.0001; Fig. 3A and FDR *P* < 0.05; Fig. 3B, respectively). On the other hand, the relative abundance of an unclassified genus in the family Lachnospiraceae was decreased in LDCD-treated mice (FDR *P* < 0.05; Fig. 3C).

The results of the multiple regression model indicated that LDCD treatment alone most impacted the results of the transit time and tail suspension tests while an interaction between LDCD and Abx treatment was observed in the cylinder test (Fig. 1). Therefore, we decided to test whether levels of these differentially abundant bacterial genera correlated with the results from these specific tests through Spearman rank-sum correlation tests (Fig. 3D; Fig. S2). *Turicibacter* levels in both the feces and ileum appeared to negatively correlate with transit time (*P* < 0.05 in feces, Fig. S2A; *P*<0.001 in ileum, Fig. S2C) indicating an association with LDD-induced improved GI motility. *Turicibacter* levels also negatively correlated with time immobile in the tail suspension test (*P* < 0.05 in feces, Fig. S2B; trending in the ileum, Fig. S2D). Fecal levels of the unclassified genus in Lachnospiraceae were not correlated with any of these tests indicating that this taxon is likely not associated with the GI and behavioral alterations induced by LDCD treatment.

To further probe whether the effects of LDCD treatment may be mediated by the microbiota, we compared the microbiota composition between mice treated with antibiotics alone (Abx) and mice treated with both LDCD and antibiotics (LDCD + Abx) using DESeq2. Interestingly, only levels of *Turicibacter* in the ileum appeared to be significantly different between these treatment groups (Fig. 3E). Ileal *Turicibacter* levels also positively correlated with improved performance in the cylinder test (*P* < 0.05, Fig. S2E), suggesting that this genus may mediate the interaction effect of LDCD and antibiotics on behavior.

To investigate the potential functional implications of LDCD-induced microbiota changes, PICRUSt2 analysis was performed to predict metabolic pathway enrichments. First, MetaCyc pathways and KO terms that have been implicated as altered in the human PD gut microbiome (24) were analyzed to see whether they were also altered in mice treated with LDCD (Fig. 3F). Bacterial metabolic pathways increased in LDCD-treated mice were involved in neuroactive signaling (dopamine, glutamate, and serotonin synthesis) and short-chain fatty acid (SCFA) production. Conversely, pathways decreased in LDCD-treated mice included one involving curli production [which has been shown to promote alpha-synuclein aggregation (25, 26)], those involved with the production of immunogenic components [lipopolysaccharide (LPS), bacterial lipoprotein (BLP), and lipoteichoic acid (LTA)], a glutamate/GABA degradation pathway, and protein degradation pathways. These pathway enrichments were similar between the fecal and ileal samples although much more prominent in the ileal samples, indicating that this might be the site of the most functional differences induced by LDCD treatment. The altered pathways appeared to indicate that LDCD treatment induced a healthier gut environment, by generally decreasing PD-associated metabolic pathways and increasing control-associated metabolic pathways.

We next looked deeper at SCFA production pathways and found that LDCD treatment appeared to increase SCFA production, particularly in the case of butyrate (Fig. 3G). The altered pathways also trended toward correlation with ileal bacterial levels in LDCD-treated mice (Fig. S3), validating that the overgrowth of bacteria at this site is associated with positive environmental changes. Altogether, these results suggest that LDCD treatment may be modifying the gut microbiota to promote an anti-inflammatory and SCFA-rich gut environment, which may lead to improvements in GI function and behavior that were observed for this treatment group.

### Antibiotic-mediated alterations in GI and motor PD-like phenotype are associated with specific clusters of bacterial genera

**Fig 4:**
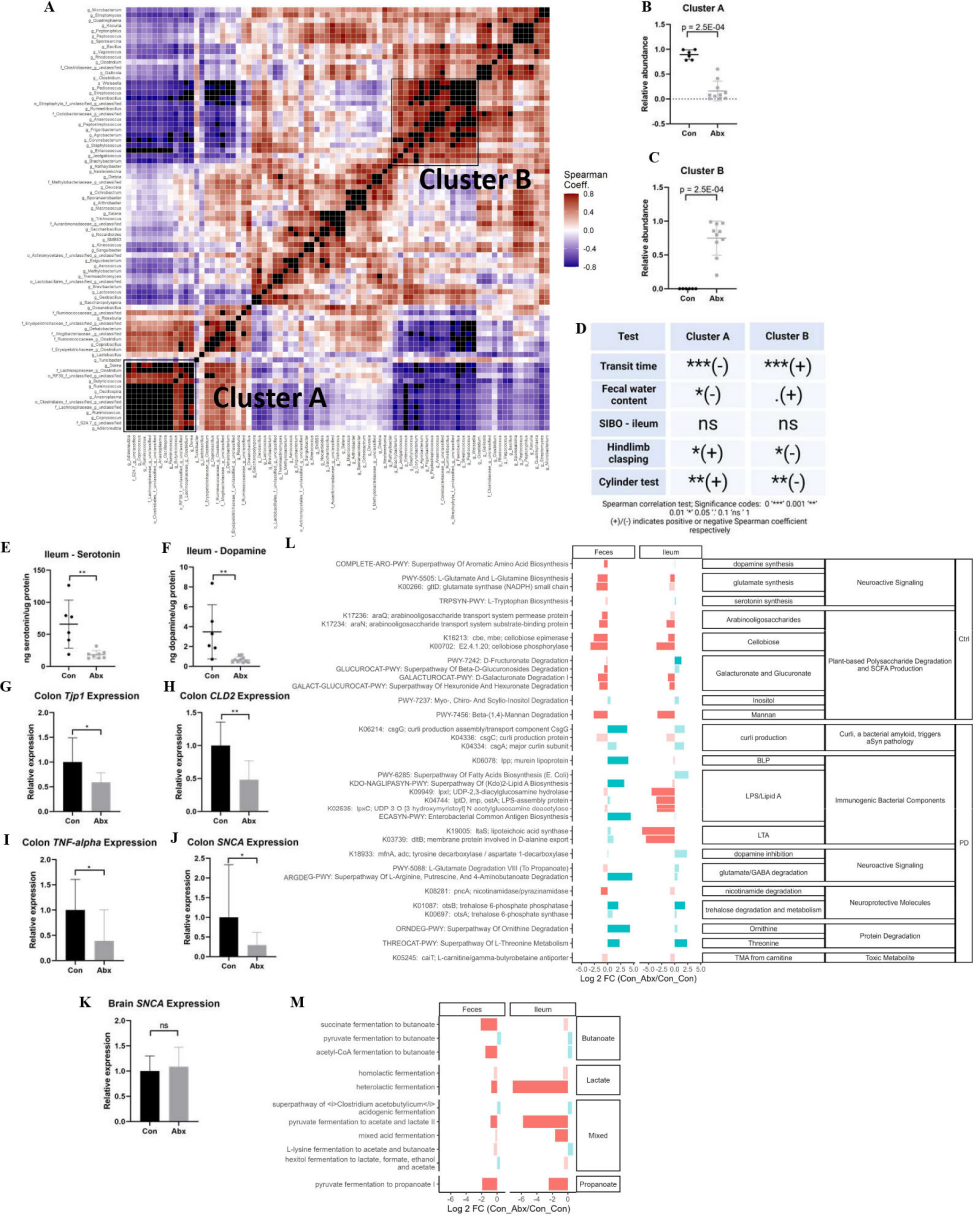
Antibiotic-mediated alterations on gastrointestinal and motor PD phenotype are associated with clusters of specific bacterial genera. (**A**) Heatmap showing covariance of bacterial genera in fecal samples of control and antibiotic-treated mice. Relative abundance of cluster A (**B**) and cluster B (**C**) in fecal samples. (**D**) Correlations between covariant cluster abundance and phenotypic tests (by Spearman correlation test, ± symbols indicate a positive or negative correlation coefficient, respectively). Ileal (**E**), serotonin, and (**F**) dopamine levels as determined by ELISA and normalized to protein content. Relative expression of (**G**) Tjp1, (**H**) CLD2, (**I**) TNF-alpha, (**J**) SNCA mRNA in the colon, and (**K**) SNCA mRNA in the brain. (**L**) PICRUSt2-predicted functional pathways in the feces and ileum of Abx mice compared to controls; solid colored bars indicate significant hits (*P*-adj <0.01 and log 2 fold change >2); blue bars indicate pathways increased in Abx mice, red bars indicated pathways increased in Con mice. Pathways are grouped by their overarching function; the rightmost column denotes which pathways are associated with PD versus controls (Ctrl) by Wallen et al. (24). (**M**) SCFA-related PICRUst2-predicted functional pathways in the feces and ileum of Abx mice compared to controls. All data are from post-treatment timepoint and two experimental replicates. Data points indicate individual mice; error bars indicate the standard deviation of the mean. *P < 0.05; **P < 0.01; ***P < 0.001; ****P < 0.0001 (by Mann-Whitney U test unless otherwise indicated). TG, transgenic; PD, Parkinson’s disease; Con, control; LDCD, L-DOPA plus carbidopa; Abx, antibiotics; SIBO, small intestinal bacterial overgrowth; SCFA, short-chain fatty acid; aSyn, alpha-synuclein; BLP, bacterial lipoprotein; LPS, lipopolysaccharide; LTA, lipoteichoic acid; GABA, γ-aminobutyric acid; TMA, trimethylamine.

Comparison of fecal and ileal microbiota composition between control and Abx-treated mice revealed that many bacterial families were altered due to treatment (Table S1 and S2), although the changes in fecal samples were more pronounced. To summarize these changes, we created a heatmap that showed clusters of covariant fecal genera (Fig. 4A). Two clusters of genera (Cluster A and Cluster B) had a mean Spearman correlation coefficient above 0.7. Indeed, Cluster A was significantly more represented in control mice (*P* < 0.001; Fig. 4B) while Cluster B was significantly more represented in Abx-treated mice (*P* < 0.001; Fig. 4C). Both clusters showed significant correlations with phenotypic tests but in opposite directions (Fig. 4D). While transit time showed a highly significant correlation with both clusters, inspection of the correlation plots (Fig. S3A and B) shows that this appears to be due to the large discrepancy in both transit time and cluster abundance between the two treatment groups. Since the correlation trends largely did not reproduce within each treatment group, a true association between these clusters and transit time is unlikely. However, two genera within Cluster A, *Anaeroplasma* and *Dorea*, showed a strong trend toward correlation with transit time in control mice alone (*P* = 0.05 for both; Fig. S3C and D), indicating that these specific genera may be related to GI motility within this PD mouse model. On the other hand, both clusters showed consistent (i.e., trends replicated within each treatment alone) correlation with the number of rearing events in the cylinder test (*P* < 0.01 for both; Fig. S3E and F) and the hindlimb clasping reflex score (*P* < 0.01 (Cluster A) and *P* < 0.05 (Cluster B); Fig. S3G and H). This suggests that the genera in Clusters A and B may be functionally linked to the motor and behavioral alterations induced by Abx treatment.

Abx treatment also resulted in a decrease in both serotonin and dopamine within the ileum (*P* < 0.01 for both; Fig. 4E and F), as well as a decrease in expression of tight junction protein 1 (*Tjp1*, *P* < 0.05; Fig. 4G) and claudin 2 (*Cldn2*, *P* < 0.01; Fig. 4H) in the colon. This indicates dysfunction within GI signaling and barrier function that could result in the increased transit time and fecal water content observed in Abx-treated mice. Interestingly, however, tumor necrosis factor-alpha (*TNF-alpha*) and alpha-synuclein (*SNCA*) transgene expression also appeared to decrease in the Abx-treated mouse colon (*P* < 0.05 for both; Fig. 4I and J) indicating a potential beneficial effect of treatment on the GI environment. *SNCA* expression levels in the brain, however, were not altered due to Abx treatment (Fig. 4K). These changes in expression of *Cldn2*, *SNCA*, and *TNF-alpha* appear to correlate with levels of either Cluster A, Cluster B, or both (Fig. S5A through E), demonstrating further evidence that these groups of taxa may be associated with the functional changes in the GI environment.

We next investigated the potential functional implications of Abx-induced microbiota alterations through PICRUSt2 analysis (Fig. 4L and M). Abx treatment induced significant changes in pathways in both fecal samples and ileal samples. In general, Abx treatment appeared to decrease pathways related to SCFA production at both sites and increase PD-associated pathways in fecal samples, potentially leading to the reduced tight junction protein expression and increased transit time observed for this treatment group. Interestingly, in ileal samples, Abx treatment seemed to decrease pathways involved in the production of immunogenic bacterial components, perhaps resulting in the decreased *TNF-alpha* and *SNCA* expression observed. The ileal (but not fecal) altered pathways were also significantly correlated with ileal bacterial levels in Abx-treated mice (Fig. S6), demonstrating that the Abx-induced loss of ileal bacteria is likely the cause of the functional pathway differences. On the whole, the functional prediction is consistent with the phenotypic and molecular changes associated with Abx treatment.

## DISCUSSION

This study suggests that long-term exposure to PD medications may have a positive impact on non-motor symptoms, potentially through beneficial alterations to the gut environment including increased *Turicibacter* abundance and butyrate production. While LDCD treatment is primarily used to improve PD motor deficits, in this study no effects on striatal function were observed. This is likely due to the short half-life of LD in circulation and the variable timing of dosage in this study as mice were able to consume LDCD water *ad libitum*. Therefore, no acute effect on motor ability was expected with this treatment regime, and our study was much better suited to examine the long-term effects of the drugs that could be potentially mediated by the gut microbiota.

We found that ileal *Turicibacter* abundance was associated with improved GI function and behavior. However, the bacterial family Turicibacteraceae has been associated with dementia-prone mice (27). *Turicibacter* has also been associated with firmer stool (a marker of constipation) in healthy female subjects (28) and has been shown to increase in abundance after serotonin administration in mice (29). *Turicibacter* levels are increased in toxin-based PD rodent models (30, 31) and PD patients in one study (24), but decreased in another human study (32). In a study of Chinese PD patients, *Turicibacter* was found to be positively associated with LD treatment initiation and negatively associated with depression (33), matching what was observed in this work. However, other studies have found increased *Turicibacter* abundance in people with depression (34). *Turicibacter* has been associated with the production of butyrate (35), an SCFA that can accelerate GI transit (36, 37). Indeed, our results indicate that LDCD treatment increases both ileal *Turicibacter* relative abundance and butyrate production pathways. Butyrate has itself been associated with positive effects on mood and depression (38). Overall, data on the role *Turicibacter* plays in PD (and specifically in the context of PD-related non-motor symptoms) are inconsistent. Our study lends evidence toward a beneficial effect of *Turicibacter* on constipation and mood in a PD context, potentially through butyrate production in the ileum.

While the data in humans lean toward negative side effects of LD on GI function and mood (8–13), our data indicate the opposite. These inconsistencies may be due to the long-term treatment explored in this study (as opposed to potential short-term side effects), or the inherent biological differences between mice and humans.

Healthy rats treated with dopamine agonists (in combination with LD and CD) showed reduced small intestinal motility as well as SIBO (39). Although whole-gut transit time was decreased in LDCD-treated mice in our study, small intestinal motility itself was not measured directly; therefore, it is possible that while overall gut motility increased, small intestinal motility could be decreased, leading toward SIBO (40). SIBO has been historically defined as the growth of more than 10^5^ CFU/mL in the proximal small intestine by culture-based techniques (41). Here, we used qPCR to quantify bacterial growth in both the jejunum and ileum to circumvent bias due to the variable culturability of different bacteria. Therefore, the small intestinal bacterial counts shown here might not be directly comparable; however, if the bacterial levels of control mice are taken as “normal,” we do observe a trend toward SIBO in LDCD-treated mice, although this only reaches significance in the distal small intestine. Nevertheless, we can effectively conclude that LDCD treatment appears to increase small intestinal bacterial growth to a certain extent. SIBO is more prevalent in PD patients (42, 43) and has been linked to negative consequences including fat/carbohydrate malabsorption, intestinal permeability, and diarrhea (41). However, the positive associations between SIBO levels and beneficial predicted functional pathways observed here indicate the opposite. It may be that the SIBO we observed in LDCD-treated mice is due to the increased abundance of *Turicibacter*, while SIBO in humans is likely composed of different genera with different functional implications.

Rats treated with various PD medications displayed microbiota alterations consistent with those reported in human PD microbiota studies; namely, increased *Lactobacillus* and *Bifidobacterium* and decreased Lachnospiraceae and Prevotellaceae, although which specific drugs induced these changes was not explored (39). These results are consistent with our findings where LDCD treatment resulted in decreased levels of Lachnospiraceae. Lachnospiraceae is consistently underrepresented in fecal samples from PD patients (16); however, this study and others (39, 44) demonstrate that this may be an artifact of the medications taken by many of the patients involved in these studies. Another study using a 6-OHDA rat model of PD found differing LD-induced microbiota alterations than those presented here (45), although this discrepancy might be due to their use of intraperitoneal injection to administer the LD treatment. In a rotenone PD mouse model, dietary intervention combined with LD treatment had an additive beneficial effect on motor function (46). While their study did not look at microbiota changes from these two interventions, since diet is known to alter the gut microbiota, these results support an interaction between the microbiota and the effects of LD.

Chronic oral treatment with vancomycin and neomycin (Abx) was found to worsen the GI PD-like phenotype in this model, exhibited by decreased GI motility but also increased fecal water content approaching diarrhea (47). The mechanism behind this Abx-induced GI dysfunction is unclear from the data obtained but some hypotheses are apparent. It may be that Abx-induced dysbiosis decreased the expression of enteric tight junction proteins leading toward a phenotype similar to exudative diarrhea or diarrhea due to motility disturbances (40). Importantly, diarrhea can result from both increasing and decreasing gut motility (40); therefore, decreased gut motility may not necessarily lead to firmer stool, as shown in our study. Genera decreased in Abx-treated mice (i.e., Cluster A) include Lachnospiraceae genera, *Oscillospira*, and *Anaeroplasma*. Lachnospiraceae are known producers of butyrate and other SCFAs (48) which have a positive impact on the gut environment (49, 50). *Anaeroplasma* (family: Anaeroplasmataceae) and *Dorea* (family: Lachnospiraceae) were found in our study to correlate with transit time. This is a relatively novel association by our understanding but may be linked to an Abx-induced decrease in SCFA production which can lead to increased colonic transit time (50). Similarly, the beneficial associations between Cluster A taxa and performance in the cylinder test may be linked to SCFA production, as these molecules have been shown to have beneficial neuroactivity (51).

Bacterial genera associated with Abx treatment (i.e., Cluster B) include *Enterococcus*, *Streptococcus*, and *Staphylococcus*. Species from these three genera have been classified as pathobionts or opportunistic pathogens (52, 53) and are related to life-threatening antibiotic resistance. Specifically, vancomycin-resistant enterococci have been reported; therefore, the increase in *Enterococcus* following vancomycin treatment found here is not unexpected. Nevertheless, the increase in these potentially harmful taxa may be linked to the negative impact observed on gut motility and barrier integrity in Abx-treated mice.

The Abx treatment here effectively decreased ileal bacterial levels, as expected given that neomycin is sometimes used to treat SIBO (41). SIBO in PD patients has been associated with worse motor function and fluctuations (43, 54), and in our study, Abx-treated mice correspondingly displayed slightly improved motor function. However, Abx-treated mice also demonstrated lower production of immunogenic components and *TNF-alpha* expression; if this anti-inflammatory environment extends to the brain, this could also lead to the improved motor function displayed by these mice.

While previous work examining the effect of a cocktail of antibiotics (which effectively deplete the gut microbiota) on motor function in this same mouse model found no significant benefit (23), this same antibiotic treatment in a different transgenic PD mouse model did lead to motor improvements (17). Given that the two antibiotics used in this study (vancomycin and neomycin) were part of both treatment regimens in the two cited studies, but the microbiota differences induced by the treatments were quite different, this is added evidence that the specific composition of the microbiota may be important in the modulation of PD motor symptoms. Indeed, the clusters of covariant bacterial genera most affected by antibiotic treatment both correlated with the improvement in motor function (as measured by the hindlimb clasping reflex test).

Vancomycin pre-treatment in an MPTP mouse model of PD was found to improve motor function potentially through microbiota alterations and the suppression of inflammation (55) and a similar mechanism may be in play here. Doxycycline followed by LD treatment of a 6-OHDA PD mouse model resulted in reduced L-DOPA-induced dyskinesia—without affecting LD-induced motor improvements—potentially by suppressing inflammation (56). Although our study used a different antibiotic treatment and PD mouse model, we also observed a reduction in *TNF-alpha* expression, as well as some mild motor improvements, due to Abx treatment. Oral vancomycin treatment has also been found to decrease levels of p-cresol sulfate (57) [a toxic microbially-produced metabolite (58)]. Interestingly, this metabolite is increased in human PD patients and associated with the bacterial genera *Oscillospira* and *Ruminococcus* (among others) (59), both of which are members of Cluster A (shown here to be decreased by Abx treatment). Therefore, our study suggests that these vancomycin-susceptible taxa may be linked to p-cresol production. Although p-cresol has not been linked to any changes in motor function, the significant correlations between Abx-altered clusters of bacteria and performance in the hindlimb clasping test suggest a possible link between these bacterial genera and motor ability.

Since both the PD medications and antibiotics in this study were administered *via* drinking water, one study limitation is the potential for large variability in dosage between experimental subjects. Only one motor test (hindlimb clasping) was performed due to an effect of treatments on motor function not being expected; however, Abx treatment appeared to have a positive effect on motor function. Unfortunately, this resulted in more robust conclusions on the effect of Abx treatment on motor function (as could be determined by a panel of motor tests) not being possible. The use of 16S sequencing to profile the gut microbiota is also not ideal as this technique is known to have a bias on multiple levels (DNA extraction differences, 16S copy numbers, and dependency on relative abundance measurements). Furthermore, we used 16S microbiota data to predict functional capabilities (through PICRUSt2) although metagenomic sequencing would undoubtedly be a more effective and robust method to obtain this information. Male mice were used exclusively in this study due to differences in availability and statistical power considerations; however, this limitation should be addressed in future studies and this work would benefit from an investigation into potential sex differences in the effects of PD medications and antibiotics. Importantly, the data presented here are purely associative, and caution should be taken in making conclusions as the correlations presented do not demonstrate a cause-and-effect relationship.

This study demonstrates that the PD medications LD and CD have beneficial effects on PD-related non-motor symptoms which may be linked to the gut microbiota genus *Turicibacter*. The finding that LDCD treatment decreases the levels of Lachnospiraceae holds implications for microbiota studies in PD patients which have consistently found this taxon to be underrepresented in the PD gut. Furthermore, we have demonstrated that treatment with antibiotics which shift but do not deplete, the gut microbiota also affects PD-related symptoms. Overall, this study reveals associations between gut bacteria and PD-like symptoms within a transgenic PD mouse model, providing insight into taxa that may be causally involved in mediating PD motor and non-motor symptoms.

## MATERIALS AND METHODS

### Animals and treatment

Dbl-PAC-Tg(*SNCA*^A53T^);*Snca*-/- transgenic mice (TG) were purchased from Jackson Laboratories (Stock no. 010799) and bred in-house to standardize the microbiota. Mice were housed in the Modified Barrier Facility at the University of British Columbia on a 12 h light-dark cycle. Ventilated cages consisted of 2–5 mice and included wood chip bedding, nesting material, and a plastic hut for enrichment. Mice had access to food [PicoLab Rodent Diet 20 - 5053 (irradiated)] and water *ad libitum*. All animal work was done in accordance with the Animal Care Committee at the University of British Columbia and the Canadian Council on Animal Care guidelines and protocols. All experiments were performed using male mice.

Treatment consisted of four experimental arms: control (Con), antibiotic-treated (Abx), PD medication-treated (LDCD), and PD medications plus antibiotics (LDCD + Abx). Abx treatment consisted of vancomycin (0.5 g/L) and neomycin (0.5 g/L) dissolved in the drinking water and administered *ad libitum*; for applicable groups of mice, abx treatment began 1 week prior—and continued during—treatment with PD medications. After 1 week of pre-treatment, L-DOPA (LD; 1 mg/mL) and carbidopa (CD; 0.25 mg/mL) were added to the drinking water of applicable groups to generate LDCD treatment. Ascorbate (2.5 mg/mL), used to prevent LD oxidation, and Splenda (4 g/L), used to promote water consumption, were added to the drinking water of all experimental groups at this stage. Treatment began when mice were in adulthood (10–15 weeks of age; see Table S3) and lasted for 4 weeks.

For experiments related to treatment with each PD medication individually, LD or CD were administered *via* drinking water in the same concentrations as outlined above. Ascorbate and Splenda were also added to the drinking water of all experimental groups as described above.

### Phenotypic testing

Male mice were tested during the light phase and in a randomized order within the same testing room during the last week of treatment. Mouse movements were recorded using a Go-PRO (Hero 6) and analyzed using ANY-maze (version 6.23, Stoelting Co.).

### Gastrointestinal testing

Whole-gut transit time was measured by the carmine red test as described previously (60) and used as an indicator of GI motility. Briefly, mice received an oral gavage of 100 uL of 6% (wt/vol) carmine red in 0.5% methylcellulose and were placed in a new clean cage containing food and water alongside cage mates. Mice were monitored every 10 min and the time elapsed from gavage to the appearance of the first red fecal pellet was recorded.

Fecal water content, used as a proxy for diarrhea, was measured as follows. Fecal pellets were collected from mice individually and stored in pre-weighed 1.5 mL microcentrifuge tubes at −20°C until further use. Tubes containing fecal pellets were weighed, lyophilized at <−20°C for 16 h, and weighed again. The percent H_2_O content was calculated by subtracting the final fecal weight from the initial fecal weight and dividing it by the initial weight.

### Motor/behavioral testing

The hindlimb clasping reflex test was used as an indicator of striatal dysfunction and motor impairment as described previously (17, 61, 62). Mice were held by the midsection of the tail and suspended for 6–8 s; the degree of clasping of the hindlimbs was recorded by video and analyzed by a blinded observer using the following criteria: 0 (no clasping) indicates flexible and freely moving hindlimbs; 1 indicates inward clasping of one hindlimb or partial inward clasping of both hindlimbs; 2 indicates inward clasping of both hindlimbs but with some flexibility; 3 indicates no flexibility and complete and immediate inward clasping of the hindlimbs.

The tail suspension test (63, 64) and cylinder test (65) were used to measure depression-like behavior and spontaneous activity respectively, as described previously (23).

### Small intestinal bacterial overgrowth

Jejunal and ileal tissue and contents were homogenized using a bead beater (FastPrep-24; MP Biomedicals). DNA was extracted from the resulting supernatant using the QIAamp PowerFecal Pro DNA kit (QIAGEN 51804) according to the manufacturer’s instructions. Quantitative PCR was performed as previously described (66) using Premix Ex Taq (Takara) and the following primers: F: 5′-CGGTGAATACGTTCYCGG-3′, R: 5′-GGWTACCTTGTTACGACTT-3′, and Probe: 5′-CTTGTACACACCGCCCGTC-3′. Serial dilutions of bacterial DNA with an established 16S copy number were used as a standard curve. The 16S copy number for each sample was obtained through comparison to the standard curve and normalized to the tissue weight.

### Serotonin and dopamine quantification

Ileal tissue (with contents) was collected, snap-frozen in liquid nitrogen, and stored at −80°C until further use. Tissue was thawed, added to 1× PBS containing 0.1% ascorbic acid (Sigma), and homogenized using tungsten beads and a Mixer Mill MM400 (Retsch; 25 Hz for 2 min). Samples were then spun on a microcentrifuge (16,000× *g*, 5 min) and the supernatant was removed for further use. Serotonin levels were determined using the Serotonin ELISA Assay kit (Eagle Biosciences EA602/96) and dopamine levels were determined using the Dopamine ELISA Assay kit (Eagle Biosciences EA608/96). The manufacturer’s instructions were followed with the following modification: for the dopamine ELISA, 150 uL of undiluted supernatant was used. Protein levels were determined using Pierce Coomassie Plus Assay Reagent (Bradford) according to the manufacturer’s instructions. Serotonin and dopamine levels were normalized to total protein content.

### Gene expression

Colon or brain tissue was collected in RNAlater (Thermo Fisher Scientific) and stored at −80°C until further use. RNA was extracted using the GeneJET RNA purification kit (Thermo Fisher Scientific), according to the manufacturer’s instructions. RNA (1 ug from each sample) was converted to cDNA using the QuantiTect Reverse Transcription kit (Qiagen), according to the manufacturer’s instructions. The QuantiTect SYBR Green PCR kit (Qiagen) was used to perform qPCR with the following sets of primers: GAPDH (reference gene) F: 5′-AAGTCGGTGTGAACGGATTTG-3′, R: 5′-TGTAGACCATGTAGTTGAGGTCA-3′; Tjp1 F: 5′-CCCTGAAAGAAGCGATTCAG-3′, R: 5′-CCCGCCTTCTGTATCTGTGT-3′; Cldn2 F: 5′-ATACTACCCTTTAGCCCTGACCGAGA-3′, R: 5′-CAGTAGGAGCACACATAACAGCTACCAC-3′; SNCA F: 5′-CAGTTGGGCAAGAATGAAGAAGG-3′, R: 5′-TCAGGTTCGTAGTCTTGATACCC-3′; TNF-alpha F: 5′-CTGTAGCCCACGTCGTAGC-3′, R: 5′-TTGAGATCCATGCCGTTG-3′.

### 16S sequencing

Fecal pellets, ileal tissue (with contents), and jejunal tissue (with contents) were collected from mice and stored at −80°C until further use. DNA was extracted using the QIAamp PowerFecal Pro DNA kit (QIAGEN 51804) according to the manufacturer’s instructions. A bead beater (FastPrep-24; MP Biomedicals) was used to homogenize fecal samples. DNA was stored at −20°C until further use. Indexed, barcoded primers (515F: GTGCCAGCMGCCGCGGTAA; 806R: GGACTACHVHHHTWTCTAAT) were used to amplify the bacterial 16S rDNA V4 region. The library was pooled and sequenced using paired-end 300-cycle reads with a v3 Reagent Kit on an Illumina MiSeq platform.

### 16S analysis

The experiment including LDCD treatment ± antibiotics and the one assessing LD or CD treatment alone were analyzed separately. Raw sequences were trimmed to 220 bp (LDCD experiment) or 200 bp (LD or CD experiment) and processed using DADA2 (67) in QIIME2 (v2019.7) (68). Alpha- and beta-diversity analyses were performed using a rarefaction depth of 2,758 (LDCD experiment) or 2,418 (LD or CD experiment). Taxonomy was assigned using the August 2013 release of Greengenes 99% OTU database. Phyloseq (69) and DESeq2 (70) in R (v4.0.2) were used to determine differential abundance. Relative abundance was used in further analysis.

Microbial covariance heatmaps were generated as described previously (59). Briefly, relative abundance data for genera above 0.1% average abundance and 10% prevalence was center log-ratio transformed, then clustered based on Spearman correlation distance and Ward linkage. Covariant genera were divided into seven clusters to result in groups containing more than five taxa each; the mean Spearman correlation coefficient was determined for each cluster, and the two most highly correlated clusters were chosen for further analyses.

DESeq2 was used to perform differential abundance on PICRUSt2-generated (71) MetaCyc pathway and KEGG Orthology (KO) data. Significant annotations were defined as having a corrected *P* value of <0.05 and an absolute log 2-fold change (Treatment/Control) of >0.585, equivalent to a 50% change from baseline. Annotations from fecal and ileal samples were plotted together to visualize any shared trends between sample types. Log 2 fold changes are represented by bar length. Trends that are not significant are displayed as partially transparent.

### Statistical analysis

In all figures, data are presented as the mean ± SD. Statistical analyses and visualizations were performed in GraphPad Prism 8 or R (v4.0.2). Multiple groups were compared by the Kruskal-Wallis test. The Mann-Whitney U test was used when comparing two groups. A two-sided *P* value < 0.05 was considered statistically significant. For microbiota analysis, Benjamini Hochberg FDR-adjusted *P* values were used for comparisons of taxonomic differential abundance between groups, beta diversity differences, and multiple hypothesis testing. Microbial taxa of interest were tested for normality using the Shapiro-Wilk test and determined to be not normally distributed. Therefore, correlations between microbial taxa and phenotypic tests were assessed through Spearman rank-sum correlation.

## Data Availability

Raw 16S amplicon sequencing reads along with their metadata were submitted to the European Nucleotide Archive (ENA) under the project code PRJEB66234.

## SUPPLEMENTAL MATERIAL

Supplemental tables and figures msphere.00379-23-s0001.docx Tables of differentially abundant bacteria between treatment groups; table of mouse treatment age ranges; figures showing treatment with individual medications, microbiota/phenotype correlations, and functional pathway correlations.

ASM does not own the copyrights to Supplemental Material that may be linked to, or accessed through, an article. The authors have granted ASM a non-exclusive, world-wide license to publish the Supplemental Material files. Please contact the corresponding author directly for reuse.
