# Supplementary material for: Microbiota-mediated effects of Parkinson’s disease medications on Parkinsonian non-motor symptoms in male transgenic mice
Source: mSphere. 2023 Dec 11;9(1):e00379-23. doi: 10.1128/msphere.00379-23 (PMC10826342; doi:10.1128/msphere.00379-23)
Supplement: Supplemental tables and figures — Tables of differentially abundant bacteria between treatment groups; table of mouse treatment age ranges; figures showing treatment with individual medications, microbiota/phenotype correlations, and functional pathway correlations. [file msphere.00379-23-s0001.docx]

Supplemental Materials

Table S1. Differentially abundant fecal bacterial families between Con and Abx-treated mice

| Significantly Different Taxa (Fecal) | Abx vs Con | |
| --- | --- | --- |
|  | Log2 Fold Change | P adj |
| f_Streptococcaceae | -8.52 | 7.79E-35 |
| f_Microbacteriaceae | -10.07 | 1.10E-26 |
| f_Leuconostocaceae | -10.59 | 1.10E-26 |
| f_mitochondria | -10.67 | 7.41E-26 |
| f_Enterococcaceae | -10.38 | 4.10E-22 |
| o_Streptophyta_f_ | -9.96 | 1.75E-20 |
| f_Staphylococcaceae | -8.15 | 4.00E-19 |
| f_Peptostreptococcaceae | -9.70 | 5.64E-18 |
| f_Paenibacillaceae | -8.13 | 5.68E-16 |
| f_Bacillaceae | -8.66 | 1.80E-15 |
| f_Tissierellaceae | -8.47 | 5.85E-13 |
| f_Corynebacteriaceae | -7.60 | 2.86E-12 |
| f_Rhizobiaceae | -7.21 | 2.43E-09 |
| f_Dermabacteraceae | -6.97 | 1.31E-08 |
| f_Planococcaceae | -8.56 | 1.14E-07 |
| f_Brevibacteriaceae | -7.03 | 1.75E-07 |
| f_S24-7 | 3.98 | 1.84E-07 |
| f_Methylobacteriaceae | -6.33 | 5.48E-07 |
| f_Sanguibacteraceae | -7.08 | 7.21E-07 |
| f_Aerococcaceae | -6.45 | 7.21E-07 |
| f_Nocardiopsaceae | -6.61 | 2.25E-06 |
| o_Lactobacillales_f_unclassified1 | -6.90 | 2.87E-06 |
| f_Actinosynnemataceae | -6.39 | 3.64E-06 |
| o_Clostridiales_f_unclassified1 | 3.14 | 6.57E-06 |
| f_Lachnospiraceae | 2.73 | 2.71E-05 |
| o_Clostridiales_f_unclassified2 | 3.27 | 2.71E-05 |
| f_Kineosporiaceae | -6.12 | 5.26E-05 |
| f_Micrococcaceae | -7.59 | 5.35E-05 |
| f_Exiguobacteraceae | -5.84 | 2.61E-04 |
| f_Ruminococcaceae | 2.48 | 5.75E-04 |
| f_Coriobacteriaceae | 2.09 | 7.17E-04 |
| f_Streptomycetaceae | -5.73 | 8.68E-04 |
| f_Anaeroplasmataceae | 3.10 | 1.30E-03 |
| f_Carnobacteriaceae | -5.49 | 4.55E-03 |
| f_Pseudonocardiaceae | -5.46 | 4.98E-03 |
| f_Mogibacteriaceae | 2.48 | 5.23E-03 |
| f_Nocardiaceae | -5.16 | 5.28E-03 |
| o_Lactobacillales_f_unclassified2 | -7.29 | 5.93E-03 |
| f_Peptococcaceae | -5.03 | 8.17E-03 |
| f_Clostridiaceae | -2.85 | 1.08E-02 |
| f_Dietziaceae | -4.84 | 2.29E-02 |
| f_Nocardioidaceae | -4.73 | 2.68E-02 |
| f_Thermoactinomycetaceae | -4.67 | 3.06E-02 |
| o_RF39_f_ | 2.19 | 4.29E-02 |
| f_Aurantimonadaceae | -4.63 | 4.89E-02 |

Table S2. Differentially abundant ileal bacterial families between Con and Abx-treated mice

| Significantly Different Taxa (Ileal) | Abx vs Con | |
| --- | --- | --- |
|  | Log2 Fold Change | P adj |
| f_S24-7_g_unclassified | 6.18 | 2.51E-14 |
| g_Adlercreutzia | 8.69 | 5.31E-07 |
| o_Clostridiales_f_unclassified1 | 5.31 | 1.74E-05 |
| f_Lachnospiraceae_g_unclassified | 5.84 | 3.59E-05 |
| g_Enterococcus | -5.07 | 1.69E-04 |
| g_Coprococcus | 6.93 | 6.58E-04 |
| g_Weissella | -4.24 | 1.11E-03 |
| o_Clostridiales_f_unclassified2 | 5.69 | 1.18E-03 |
| g_Peptostreptococcus | -6.71 | 3.11E-03 |
| f_Pseudomonadaceae_g_unclassified | -6.01 | 1.05E-02 |
| g_Clostridium | -5.58 | 1.07E-02 |
| g_Oceanobacillus | -5.81 | 1.42E-02 |
| g_Oscillospira | 5.04 | 2.08E-02 |
| g_Staphylococcus | -3.16 | 2.90E-02 |
| g_Frigoribacterium | -5.59 | 3.11E-02 |
| g_Thermoactinomyces | -5.76 | 3.11E-02 |
| g_Paenibacillus | -3.30 | 3.11E-02 |
| g_Dorea | 5.74 | 3.36E-02 |
| g_Streptococcus | -2.79 | 3.74E-02 |
| f_Aurantimonadaceae_g_unclassified | -5.28 | 4.83E-02 |

Table S3. Start of treatment ages for mice used in behavioral analyses

| Treatment Group/Mouse | Start of Treatment Age (weeks) | Treatment Group/Mouse | Start of Treatment Age (weeks) |
| --- | --- | --- | --- |
| **Con** | | **LDCD** | |
| 1 | 11 | 1 | 11 |
| 2 | 11 | 2 | 11 |
| 3 | 11 | 3 | 11 |
| 4 | 15 | 4 | 11 |
| 5 | 15 | 5 | 11 |
| 6 | 15 | 6 | 13 |
| 7 | 11 | 7 | 13 |
| 8 | 11 | 8 | 13 |
| 9 | 11 | 9 | 15 |
| 10 | 11 | 10 | 15 |
| 11 | 11 | 11 | 11 |
| 12 | 11 | 12 | 11 |
| 13 | 11 | 13 | 11 |
| 14 | 11 | 14 | 11 |
| 15 | 11 | 15 | 11 |
| 16 | 11 | 16 | 11 |
| 17 | 11 | 17 | 11 |
| 18 | 11 | 18 | 13 |
| 19 | 11 | 19 | 13 |
| 20 | 13 | 20 | 13 |
| 21 | 13 | 21 | 13 |
| 22 | 13 | 22 | 14 |
| 23 | 13 | 23 | 14 |
| 24 | 14 | 24 | 14 |
| 25 | 14 | 25 | 14 |
| 26 | 13 | 26 | 14 |
| 27 | 13 | 27 | 13 |
| 28 | 13 | 28 | 13 |
| 29 | 13 | 29 | 13 |
| 30 | 13 | 30 | 10 |
| **Abx** | | 31 | 10 |
| 1 | 12 | **LDCD+Abx** | |
| 2 | 12 | 1 | 12 |
| 3 | 12 | 2 | 12 |
| 4 | 10 | 3 | 12 |
| 5 | 10 | 4 | 10 |
| 6 | 15 | 5 | 10 |
| 7 | 15 | 6 | 10 |
| 8 | 15 | 7 | 15 |
| 9 | 13 | 8 | 15 |
| 10 | 13 | 9 | 15 |
|  | | 10 | 12 |
|  |  | 11 | 12 |
|  |  | 12 | 12 |


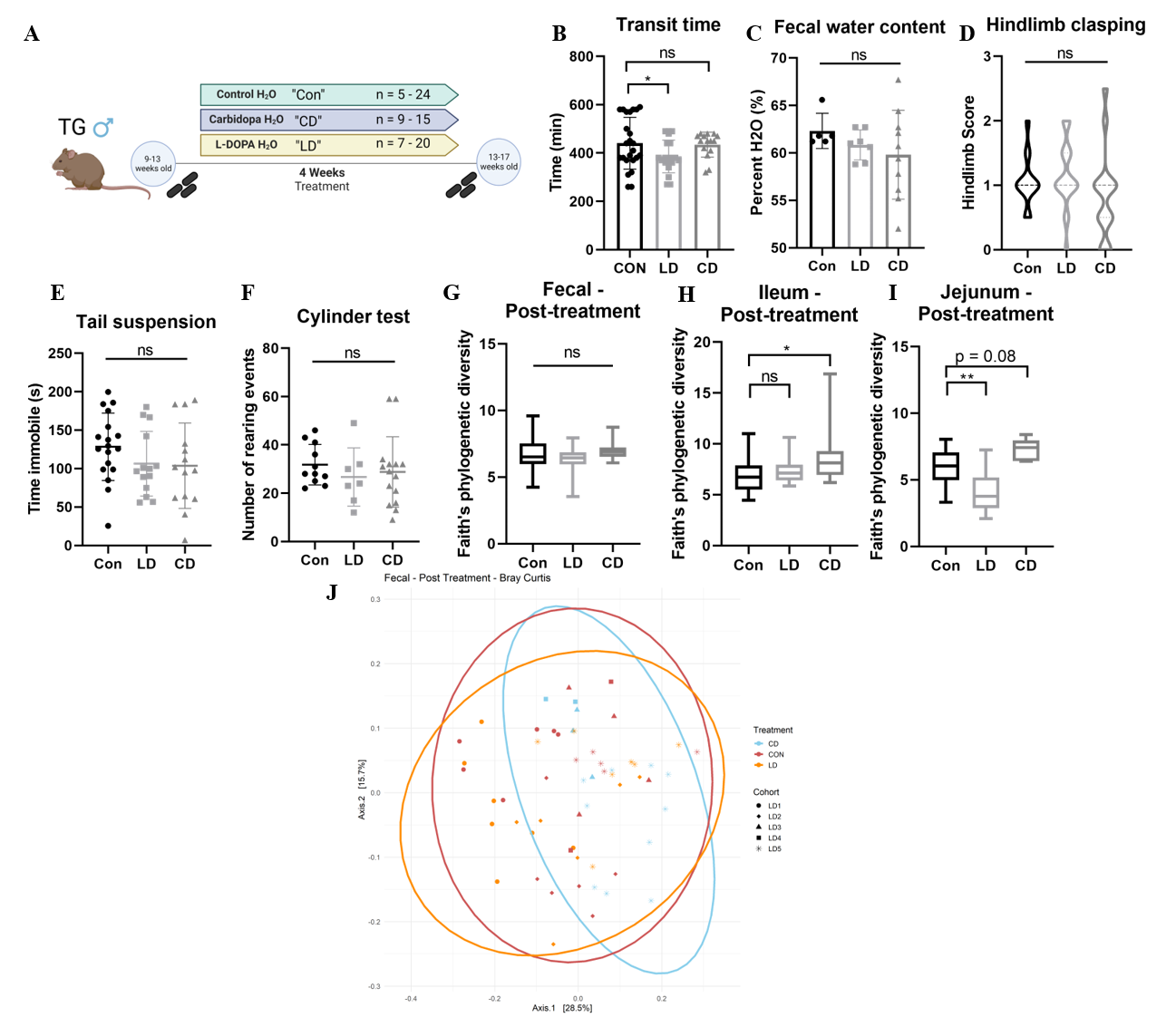


Figure S1. Parkinson’s medications individually minimally modulate the disease-associated phenotype in a PD mouse model. (A) Experimental design and timeline. (B) Whole-gut transit time. (C) Fecal water content. (D) Hindlimb clasping reflex score. (E) Time spend immobile in tail suspension test. (F) Number of rearing events in cylinder test. Alpha diversity – as measured by Faith’s phylogenetic diversity in (G) fecal, (H) ileal, and (I) jejunal samples. (J) Bray Curtis beta diversity of fecal samples post-treatment; colours indicate different treatments, shapes indicated different experimental replicates. All data are from post-treatment timepoint and from 2-5 experimental replicates. Data points indicate individual mice; error bars indicate standard deviation of the mean. ∗p < 0.05; ∗∗p < 0.01; ∗∗∗p < 0.001; ∗∗∗∗p < 0.0001 (by Mann-Whitney U test unless otherwise indicated). TG, transgenic; PD, Parkinson’s disease; Con, control; LD, L-DOPA; CD, carbidopa.


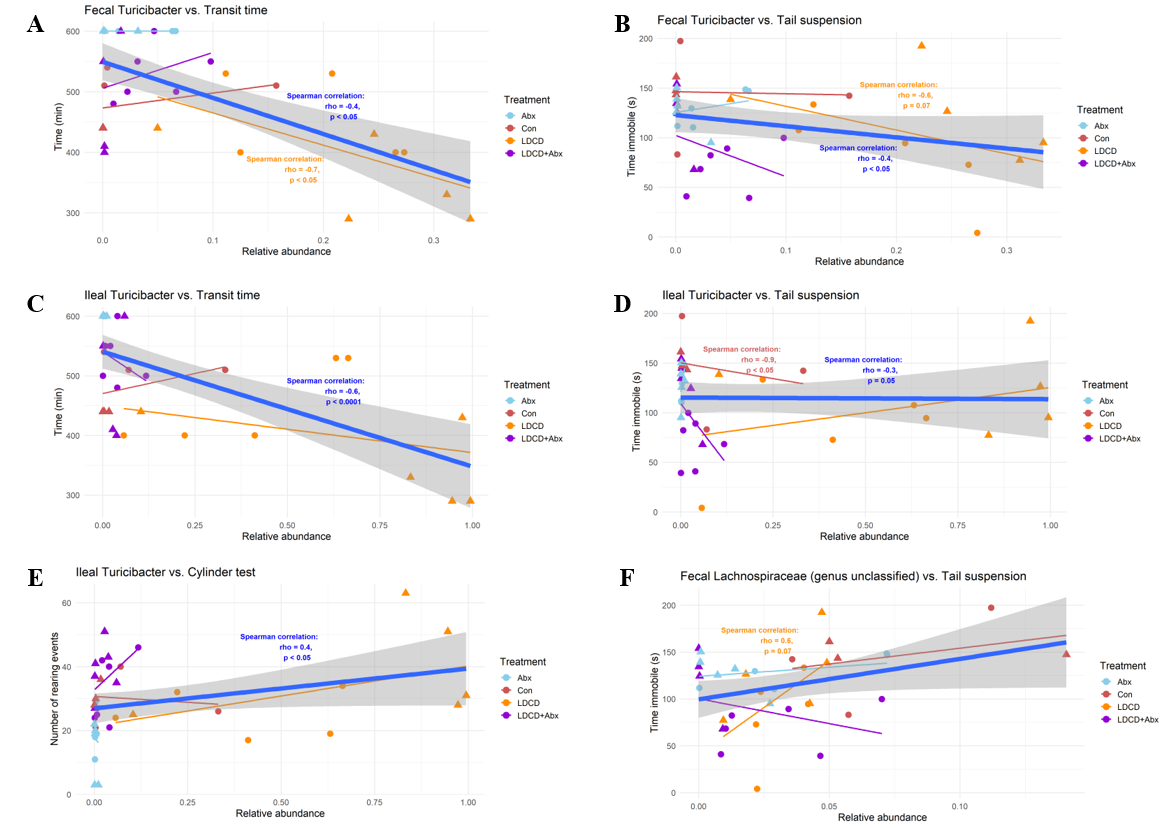


Figure S2. Correlations between bacterial genera altered by LDCD treatment and PD-related phenotypic tests, as well as enteric neurotransmitter levels. Spearman correlation between fecal Turicibacter and: (A) transit time, (B) tail suspension; ileal Turicibacter and: (C) transit time, (D) tail suspension, (E) cylinder test; fecal Lachnospiraceae (unclassified genus) and (F) tail suspension. Data points indicate individual mice; colours indicate treatment. All data is from two experimental replicates (indicated by shape of data points). Error bars indicate standard deviation of the mean. PD, Parkinson’s disease; Con, control; LDCD, L-DOPA plus carbidopa, Abx, antibiotics.


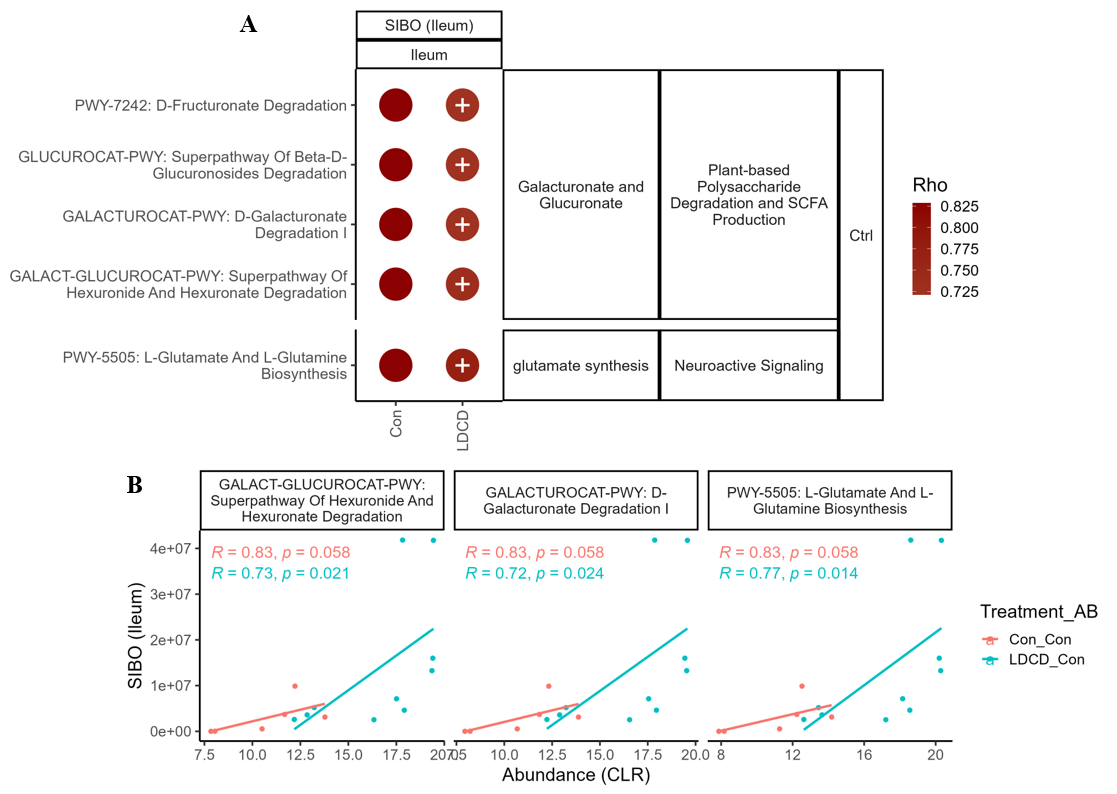


Figure S3. Ileal functional pathways altered by LDCD treatment correlate with levels of bacteria in the ileum. (A) Correlations between bacterial levels in the ileum (SIBO) and centre-log-transformed abundance of PICRUSt2-predicted functional pathways in the ileum; colour indicates Spearman correlation coefficient (rho). Pathways are grouped by their overarching function. The rightmost column denotes which pathways are associated with PD versus controls (Ctrl) by Wallen et al. (2022). (B) Representative scatter plots of correlations with pathways predicted from ileal samples. Data points indicate individual mice; colours indicate treatment. All data is from two experimental replicates. Statistics indicated are as follows: +p<0.1, *p<0.05, **p<0.01, and ***p<0.001 (FDR-corrected Spearman correlation). PD, Parkinson’s disease; Con, control; LDCD, L-DOPA plus carbidopa, SIBO, small intestinal bacterial overgrowth; SCFA, short-chain fatty acid.


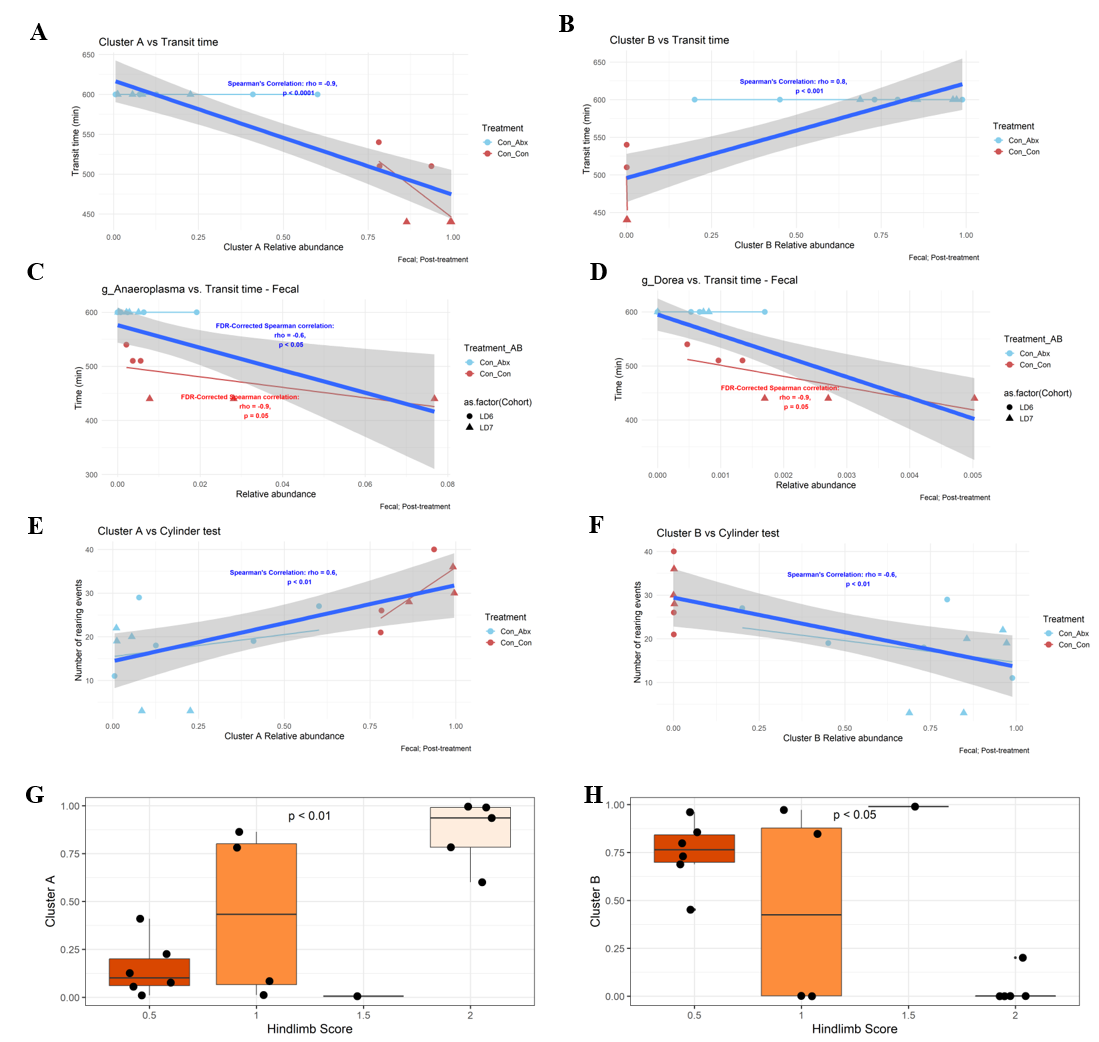


Figure S4. Correlations between clusters of covariant fecal bacterial genera altered by Abx treatment and PD-related phenotypic tests. Spearman correlation between transit time and: (A) Cluster A, (B) Cluster B, Cluster A constituents (C) Anaeroplasma and (D) Dorea. Spearman correlation between the number of rearing events in the cylinder test and: (E) Cluster A, (F) Cluster B. Overall correlation coefficients and p values (for Con and Abx samples together) are shown in blue, correlation coefficients and p values for Con mice only shown in red (FDR corrected p values used here due to testing of multiple Cluster A genera). Correlations between hindlimb clasping score and: (H) Cluster A, (G) Cluster B; statistics were determined by ordinal logistic regression due to the categorical and ordered nature of hindlimb scores, median and range are shown. Data points indicate individual mice; colours indicate treatment. All data is from two experimental replicates (indicated by shape of data points). PD, Parkinson’s disease; Con, control; LDCD, L-DOPA plus carbidopa, Abx, antibiotics.


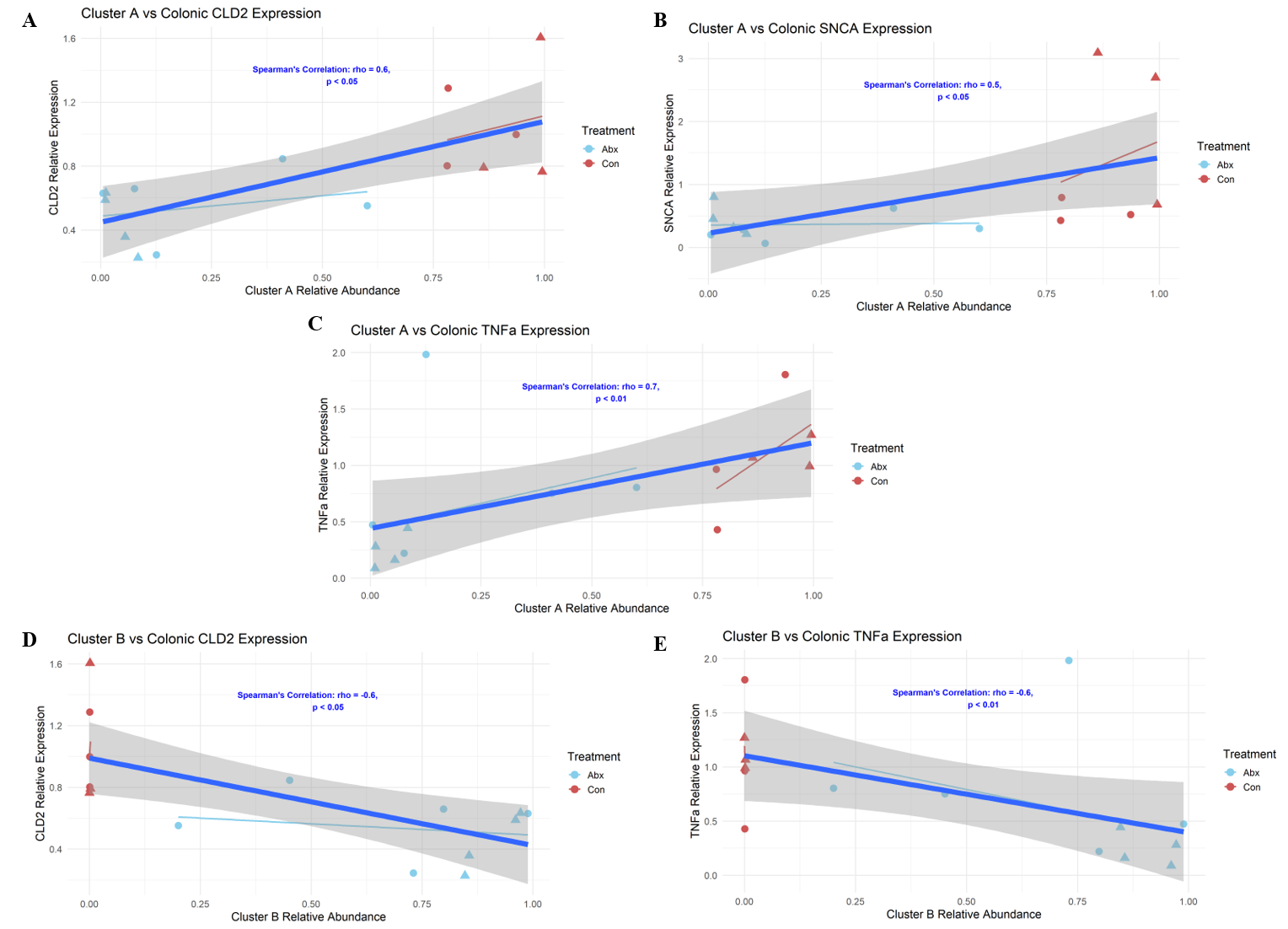


Figure S5. Correlations between clusters of covariant fecal bacterial genera altered by Abx treatment and colonic gene expression levels. Spearman correlation between Cluster A and relative expression of: (A) CLD2, (B) SNCA, and (C) Tnfa. Spearman correlation between Cluster B and relative expression of: (D) CLD2 and (E) Tnfa. Overall correlation coefficients and p values (for Con and Abx samples together) are shown in blue. Data points indicate individual mice; colours indicate treatment. All data is from two experimental replicates (indicated by shape of data points). PD, Parkinson’s disease; Con, control; LDCD, L-DOPA plus carbidopa, Abx, antibiotics.


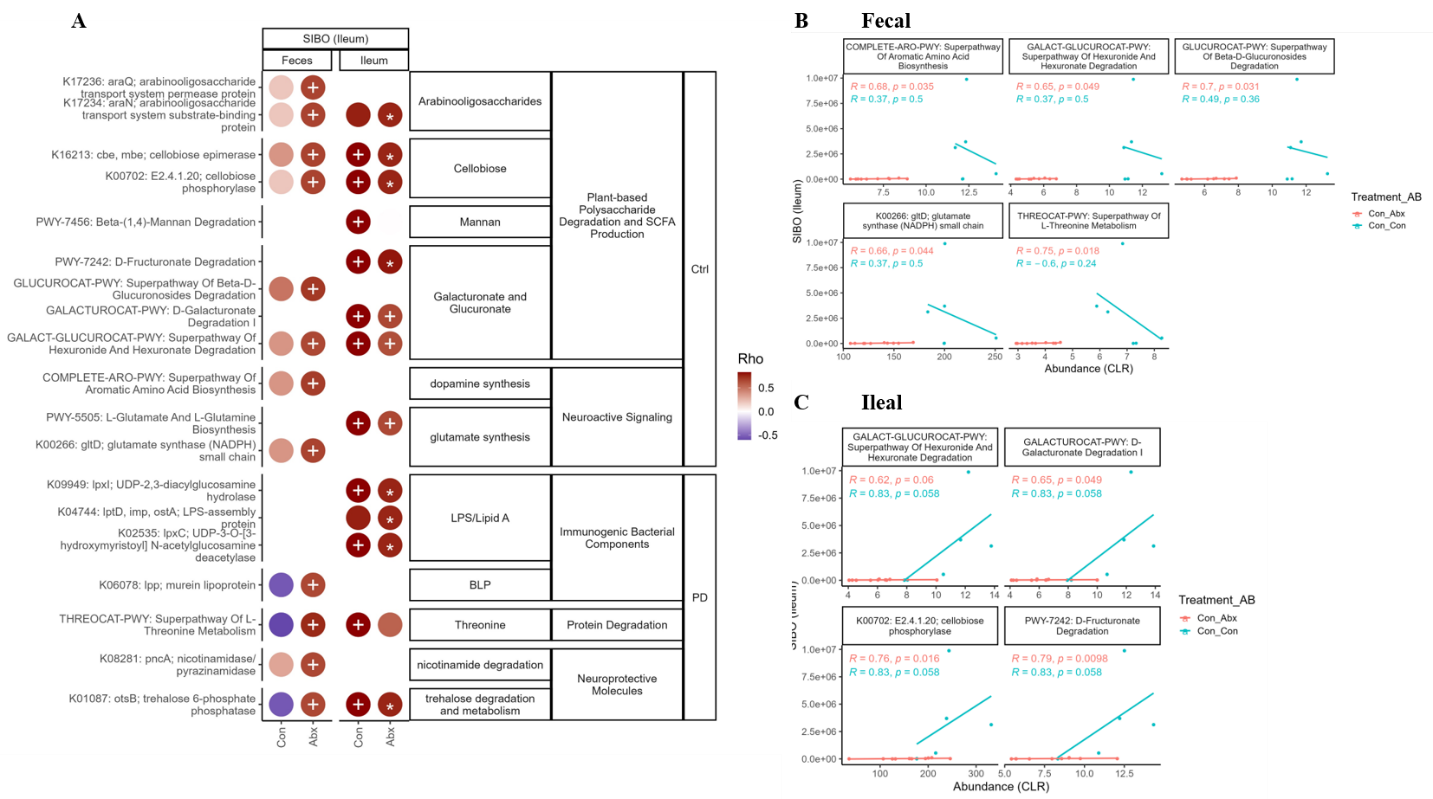


Figure S6. Ileal, but not fecal, functional pathways altered by antibiotic treatment correlate with levels of bacteria in the ileum. (A) Correlations between bacterial levels in the ileum (SIBO) and centre-log-transformed abundance of PICRUSt2-predicted functional pathways in the feces and ileum; colour indicates Spearman correlation coefficient (rho). Pathways are grouped by their overarching function. The rightmost column denotes which pathways are associated with PD versus controls (Ctrl) by Wallen et al. (2022). (B) Representative scatter plots of correlations with pathways predicted from fecal samples. (C) Representative scatter plots of correlations with pathways predicted from ileal samples. Data points indicate individual mice; colours indicate treatment. All data is from two experimental replicates. Statistics indicated are as follows: +p<0.1, *p<0.05, **p<0.01, and ***p<0.001 (FDR-corrected Spearman correlation). PD, Parkinson’s disease; Con, control; Abx, antibiotics; SIBO, small intestinal bacterial overgrowth; SCFA, short-chain fatty acid; LPS, lipopolysaccharide; BLP, bacterial lipoproteins.
